# Diseases of the Intestines

**Published:** 1905-08-05

**Authors:** 


### Progress in Medicine and Surgery.

### DISEASES OF THE INTESTINES.

(*Continued from page* 309.)

**Intestinal Peristalsis and Purgatives.** — The rhythmical contractions of the intestine have been shown by Magnus[8] to have a nervous, and not a muscular origin. By a series of experiments on the intestines of the cat, he shows that the integrity of Auerbach's plexus is necessary in order to start peristalsis, but that once started the movement may be transmitted without either Auerbach's or Meissner's plexus. He also shows reasons for believing that the impulse when started in the nerve centres is not transmitted along the muscle fibres, but along the fine nerve plexus between the cells, which thus appears to be a purely conducting mechanism, having no power of exciting the wave of contraction. Magnus has also shown that there is a refractory period during the contraction-wave of the cat's intestine lasting about five seconds, or longer if the temperature is lowered. This phenomenon is dependent on the nerve centres; in the muscle itself, freed from the nerve plexus, no refractory period occurs, a succession of stimuli producing eventually a true tetanic contraction. In connection with this subject Macallum's[9] experiments on the action of purgatives are of interest. He experimented with three classes of drugs, salines, vegetable purges (such as rhubarb and senna), and alkaloids (such as pilocarpine). He found that in the case of salines the theory that they act by attracting a large amount of fluid secretion into the intestine in the process of absorption can no longer be held. These salts, when injected directly into the blood, act more promptly than when introduced into the bowel, 1 to 2 cc. of $n/8$ solution of sodium citrate producing an almost immediate effect. He believes, therefore, that the action of saline purges is due to their power of producing increased excitability of muscles and possibly nerves, leading to increased peristalsis and increased secretion, and that this action only takes place after the saline has been absorbed into the blood-stream. He finds, moreover, that this action is neutralised by calcium or magnesium chloride. The action of the vegetable purges is similar, but the inhibitory effect of the above-named salts is transitory and soon passes off, while with pilocarpine, the antagonism is even less marked.

**Appendicitis.**—The supposed increase of cases of appendicitis is due, MacFarland[10] thinks, to improved methods of diagnosis. He considers that surgeons who see mainly the most severe cases are apt to overlook the great majority of mild cases which do not need operation. He does not think that the majority of cases need operating on in the acute stage, and states that some mortality is inevitable under any line of treatment, though personally he has never lost a case. Ninety to ninety-five per cent. of acute cases recover without operation and not more than 25 per cent. relapse. The best surgeons have a mortality of five per cent. He advises absolute rest, locally an ice-bag, the withholding of all food except a little sweetened water, and opium suppositories. The bowels should not be opened for a week or 10 days. The rectum should always be examined, especially in women, and a leucocyte count taken. If there is a low count on the fourth day operation is contra-indicated. Operation during the quiescent period is practically free from mortality. Macewen[11] has suggested that the chief function of the appendix is to cultivate an attenuated strain of the B. Coli, with which it inoculates the cæcal contents. He has observed in a case of cæcal fistula a thick alkaline fluid poured out from the appendix and cæcal glands just before the acid chyme passed through the ileo-cæcal valve. When the contents of the cæcum became too acid the skin and mucous membrane were digested by the acid ferments in the chyme.

**Diagnosis of Abdominal Conditions.**—A case is published by Barnett,[12] diagnosed as dysentery, which proved at the autopsy to be a chronic intussusception. The patient was a soldier aged 24 years, who had been suffering for some months from constipation and the passage of blood from the rectum. He had previously in South Africa been in hospital with dysentery and later with ulceration of the rectum. Barnett found no rectal ulcers on examination; the patient passed a number of fluid stools containing clots and some which were pure blood. There was also pain and tenesmus. He was treated for dysentery and after two months, though improved locally he failed to gain in weight and eventually died with symptoms of acute obstruction. There was no ulceration found post-mortem but the ileum was the seat of a chronic intussusception for the last 10 inches of its length. Another rare condition is recorded by Richardson[13] in a boy aged 11 years. For more than 12 months he had suffered from attacks of acute indigestion and the physical signs of fluid in the abdominal cavity. There was some pyrexia, the boy was cachectic, and the abdomen was very tense and prominent. Tuberculous peritonitis was diagnosed, but at the operation a large chylous cyst was found behind the peritoneum containing about a gallon of albuminous milky fluid. It was dissected out and removed, but its exact origin could not be determined. Recovery was rapid.

**Mucomembranous Enteritis.**— Three cases of this condition have been described by Vinay[14] apparently due to traumatism. The first patient was a man of 24, and the condition was started by a violent effort to draw a cork. A sudden acute pain over the cæcum and ascending colon occurred, followed by digestive troubles, the passage of mucus and blood, and increased acidity, uric acid and urea in the urine. False membranes were occasionally passed, and the patient was definitely neurasthenic. In the second case, that of a man, aged 44, the acute pain followed a violent effort to move a pump-handle. This was followed by a catarrhal diarrhœa, paroxysmal pains, and later the passage of false membranes. There were also symptoms of enteroptosis and neurasthenia. The third case occurred in a woman aged 25. Three years previously she had fallen and bruised the left side. She had sharp pain over the ascending colon and sigmoid, with the passage of membranes and blood. This condition recurred on exertion, but was improved by rest, dieting, and olive-oil injections through a long tube. Vinay considers the accidents too slight to account for the local condition, which he believes was reflexly produced by injury to the solar plexus. He classes all three cases as examples of traumatic neurasthenia.

**Chronic Constipation.**—Constipation due to atony of the colon is supposed to be due to errors in diet.[15] Soft food hastily swallowed fails to irritate the colon sufficiently to cause it to expel its contents. A condition of auto-intoxication is set up which gradually increases in intensity, until the transverse colon is filled with solid masses, leading to stercoral ulceration and colitis, and perhaps to localised peritonitis binding down and constricting the colon. Lane has proposed in these cases, where medical measures are useless, to short-circuit the bowel by uniting the lower end of the ileum to the lower portion of the colon. Grey[16] has described a case in a boy aged thirteen and a half years which yielded to medical treatment. For eleven years his bowels had only acted every three to six weeks, and once he had gone as long as two months without an action. In addition to local symptoms his physical and mental development was much retarded. Grey found the descending colon imperfectly developed, and considered the condition partly due to spasm of the sphincter ani. He overcame the spasm by means of cocaine and morphia suppositories, and then was able to remove the fæcal matter by injections of hot olive oil, 30 oz. being used twice daily. In one week he removed 26¼ lb. of solid matter; after nine days the fæces became more normal in character, and then with a proper dietary, massage and exercises, and a judicious use of purgatives, a normal condition was attained. The patient's mental and physical development then rapidly improved.

[8] Edin. Med. Jour., 1905, p. 280. [9] Ibid., Dec. 1904, p. 552. [10] Med. Rec., Feb. 25, 1905, p. 298. [11] Lancet, Dec. 24, 1904, p. 1796. [12] Ibid., Dec. 10, 1904, p. 1665. [13] Ibid., March 4, 1905, p. 587. [14] Brit. Med. Jour. Epit., Dec. 24, 1904, No. 360. [15] Lancet, Dec. 24, 1904, p. 1796. [16] Med. Rec., March 4, 1905, p. 249.

